# Changes in Body Composition Compartments After Kidney Transplantation: A One-Year Prospective Study

**DOI:** 10.3390/jcm14207131

**Published:** 2025-10-10

**Authors:** Emilia Ferrer-López, Raúl López-Blasco, Francisco Javier Rubio-Castañeda, Víctor Cantín-Lahoz, Juan José Aguilón-Leiva, María García-Magán, Carlos Navas-Ferrer, Isabel Blázquez-Ornat, María Teresa Fernández-Rodrigo, Isabel Antón-Solanas, Fernando Urcola-Pardo

**Affiliations:** 1 Department of Physiatry and Nursing, Faculty of Health Sciences, University of Zaragoza, C/Domingo Miral s/n, 50009 Zaragoza, Spain; eferrerl@unizar.es (E.F.-L.); jaguilon@unizar.es (J.J.A.-L.); mgmagan@unizar.es (M.G.-M.); cnavasf@unizar.es (C.N.-F.); iblazquez@unizar.es (I.B.-O.); maitefer@unizar.es (M.T.F.-R.); furcola@unizar.es (F.U.-P.); 2 SAPIENF Research Group (B53-23R), Universidad de Zaragoza, C/Pedro Cerbuna, 12, 50009 Zaragoza, Spain; 3 Haemodialysis and Renal Transplant Unit, Hospital Universitario Miguel Servet de Zaragoza, Paseo Isabel la Católica 1-3, 50009 Zaragoza, Spain; fjrubioc@salud.aragon.es (F.J.R.-C.); vcantin@salud.aragon.es (V.C.-L.); 4 GIIS073-Renal Research and Transplantation Group (GINETE), Aragón Health Research Institute (IISA), Aragón Biomedical Research Centre (CIBA), San Juan Bosco, 13, 50009 Zaragoza, Spain; 5 Biocomputing Unit, Aragon Health Sciences Institute, San Juan Bosco, 13, 50009 Zaragoza, Spain; rlopezb.iacs@aragon.es; 6 GIIS071-Urology Group, Miguel Servet University Hospital (URO-SERVET), Aragón Health Research Institute (IISA), Aragón Biomedical Research Centre (CIBA), San Juan Bosco, 13, 50009 Zaragoza, Spain

**Keywords:** kidney transplantation, body composition, weight gain, muscle mass, fat mass, visceral fat, total body water, bioelectrical impedance, cardiovascular risk factors

## Abstract

**Background/Objectives**: Weight gain after kidney transplantation is frequent but heterogeneous, often accompanied by changes in body composition that influence long-term outcomes. This study analysed one-year changes in body compartments and their demographic and clinical determinants. **Methods**: A prospective cohort of 112 adult kidney recipients transplanted between September 2020 and June 2022 at a Spanish tertiary hospital was followed. Body weight, muscle mass, fat mass, visceral fat and total body water were assessed by multi-frequency bioelectrical impedance at discharge, and at 3, 6 and 12 months. Associations with sociodemographic, clinical and comorbidity variables were examined using repeated-measures ANOVA and comparative tests. **Results**: At 12 months, mean weight gain was 3.6 ± 6.5 kg (5.1%). Increases were greater in men, younger patients, non-dialysis candidates, those with previous transplantation and living donor grafts. Muscle mass rose during the first three months and then stabilised, with greater gains in men and haemodialysis patients. Fat mass decreased initially and then increased, particularly in women, younger recipients and living donor transplants. Visceral fat progressively increased after three months, with higher levels in men and older patients. Total body water declined in women, younger recipients and first transplant patients. Patients with new-onset diabetes gained less weight, while smokers gained more. **Conclusions**: Post-transplant body composition is shaped by sex, age, BMI, comorbidities and donor type. Monitoring compartments beyond body weight may allow early detection of adverse metabolic trajectories. Tailored nutritional and lifestyle interventions are needed to optimise long-term outcomes.

## 1. Introduction

Weight gain after kidney transplantation is well documented in the literature [1,2,3,4]. Roughly half of all recipients gain weight during the first post-operative year, regardless of their pre-transplant nutritional status, and such weight gain can occasionally reflect recovery from pre-existing malnutrition [5,6,7]. While the prevalence of obesity before transplantation mirrors that of the Spanish general population (≈22%) [8,9], the figure rises to about 36% one year after graft implantation [10,11].

Unlike patients on haemodialysis—where a higher BMI appears to confer survival benefit—the same degree of adiposity is not protective in transplant recipients [12,13]. Excess fat mass, together with disturbances in glucose and lipid metabolism, volume overload and cardiac strain, contributes to a markedly elevated cardiovascular risk in this population [14,15,16]. In a retrospective UK cohort of 25,539 adult kidney-transplant recipients, a BMI > 25 kg m^−2^ independently predicted delayed graft function and primary non-function [17]. Growing evidence also links overall obesity and, more specifically, visceral adiposity with poorer graft and patient survival, even in individuals with an otherwise well-functioning transplant [2,18].

### Background

Beyond metabolic disturbances, kidney transplant recipients face significant clinical and emotional challenges. Rejection, graft function and long-term survival are primary concerns, especially considering that some patients may require more than one transplant during their lifetime, making this a clinically complex and emotionally variable population [19]. This complexity is compounded by common pre-existing comorbidities, including hypertension (HT), diabetes mellitus (DM), dyslipidaemia (DLP) and cardiovascular disease (CVD), both coronary and peripheral, as well as polypharmacy and older age [20].

Immunosuppressive therapy, essential to prevent graft rejection, increases the risk of opportunistic infections, hypertension, hypercholesterolaemia and the development of post-transplant diabetes mellitus (PTDM). Similarly, corticosteroids—especially during the early post-operative period—exert metabolic effects and stimulate appetite, contributing to weight gain [20,21,22].

At the same time, kidney transplantation resolves the anorexic and hypercatabolic state associated with end-stage renal disease by eliminating protein losses due to dialysis, improving nutritional intake through the removal of dietary restrictions, and allowing for greater freedom in daily life. These changes typically lead to psychological improvement and better quality of life [23] but also make it more difficult to control food intake and body weight [4].

However, obesity in this population is not homogeneous. Some individuals with obesity remain metabolically healthy, while others develop significant cardiometabolic comorbidities despite similar levels of adiposity [24,25]. Moreover, after transplantation, significant changes occur in body composition, including variations in muscle mass, fat redistribution and fluid balance. These changes may be driven by surgical immobilisation, the catabolic effect of corticosteroids, reduced physical activity and liberalisation of the diet [26,27].

Accordingly, maintaining weight with a favourable body composition becomes a self-care priority after kidney transplantation. If weight gain is primarily due to increased fat mass, it may serve as a risk marker for reduced graft function and survival [15,16]. In contrast, restoring healthy body composition may enhance quality of life, reduce the risk of metabolic diseases and contribute to long-term graft preservation [28].

Given the clinical impact of post-transplant weight gain and the heterogeneity of obesity phenotypes, it is essential to go beyond body weight and examine specific changes in body composition compartments. We hypothesised that body composition changes during the first year after kidney transplantation would vary according to demographic and clinical factors, with greater fat accumulation expected among recipients with cardiovascular risk factors, and more favourable trajectories among younger patients and those receiving grafts from living donors. The aim of this study was to analyse one-year changes in weight and body composition in a prospective cohort of kidney transplant recipients, and to explore their associations with cardiovascular risk factors such as hypertension, diabetes mellitus, dyslipidaemia, overweight and obesity.

## 2. Materials and Methods

### 2.1. Study Design and Participants

A descriptive, longitudinal and prospective study was conducted in kidney transplant recipients. The study population included all individuals who received a kidney graft between 1 September 2020 and 30 June 2022 in our autonomous community.

Inclusion criteria were being aged 18 years or older and having provided informed consent to participate in the study. Exclusion criteria included refusal to participate and amputation of one or more limbs during the follow-up period.

A total of 117 patients received a kidney transplant during the study period. Four were excluded due to the inability to record weight at the first post-discharge consultation, and one additional patient was excluded due to a lower limb amputation three months after transplantation. The final sample consisted of 112 participants.

### 2.2. Variables, Data Collection and Measuring Instruments

Sociodemographic variables included age and sex. Clinical variables comprised anthropometric measurements (height and weight), presence of comorbidities (hypertension, diabetes mellitus, dyslipidaemia, cardiovascular disease, cardiorespiratory disease or stroke), smoking status, time on the transplant waiting list, and type of renal replacement therapy prior to transplantation (non-dialysis, peritoneal dialysis or haemodialysis). These data were obtained from the electronic medical records of the Spanish National Health System.

In our cohort, all kidney transplants were performed between donors and recipients of the same blood group, and patients received the standard care of our autonomous community, which includes the recommendation to follow a Mediterranean diet and to walk 30 min daily after complete wound healing.

Data collection was conducted from September 2020 to July 2023. Weight and body composition were assessed at outpatient visits scheduled 7 days after discharge (baseline), and at 3, 6 and 12 months post-transplantation. All measurements were taken at the hospital’s outpatient nursing unit during nephrology follow-up appointments.

Body composition was assessed using the Tanita BC-601F segmental body composition monitor, which employs multi-frequency bioelectrical impedance analysis (BIA) with five measurement pathways (foot-to-foot, hand-to-hand, left hand to right foot, right hand to left foot, and left hand to left foot). This system covers 100% of the body surface, in contrast to conventional BIA devices that typically reach only 75%, thus enhancing accuracy in individuals with non-standard body composition profiles, such as transplant recipients.

The variables analysed included weight (kg), body fat percentage, muscle mass percentage, visceral fat index, total body water and BMI. Other parameters (e.g., limb-specific measures, bone mass, metabolic age) were excluded from analysis. Measurements were performed following the manufacturer’s recommendations: patients stood barefoot with feet centred on the scale sensors, held the handgrip bar with both hands ensuring all ten fingers contacted the electrodes, and extended their arms without touching the body. Participant age, sex and height were entered into the scale prior to each measurement. Body sizing was performed during pre-surgical preparation using MeWa GmbH equipment (Schwerin M-3040-40-01).

### 2.3. Data Analisys

Statistical analyses were performed using IBM SPSS Statistics for Windows, Version 27.0 (IBM Corp., Armonk, NY, USA). Qualitative variables were summarised using absolute and relative frequencies (*n*, %). Quantitative variables were expressed as mean and standard deviation (SD) when normally distributed, or median and interquartile range (IQR: 25th–75th percentile) when not.

Comparisons between qualitative variables were conducted using the chi-square test or Fisher’s exact test. For quantitative variables, comparisons were made using parametric tests (Student’s *t*-test, paired *t*-test, one-way ANOVA and repeated-measures ANOVA with Bonferroni post hoc correction) when assumptions of normality and sphericity were met. In the absence of normal distribution, non-parametric tests were used (Mann–Whitney U, Kruskal–Wallis and Wilcoxon signed-rank test). Pearson’s or Spearman’s correlation coefficients were applied to assess associations between continuous variables. Statistical significance was established at *p* < 0.05, with 95% confidence intervals (CI) where applicable.

### 2.4. Ethical Considerations

Prior to data collection, all participants received verbal and written information about the study and provided informed consent. The study adhered to current ethical and legal standards regarding data protection and biomedical research, in accordance with Regulation (EU) 2018/1725 of the European Parliament and of the Council, and Spanish Organic Law 3/2018 on the Protection of Personal Data and Guarantee of Digital Rights. The principles outlined in the Declaration of Helsinki [29] were also followed. Patient names, initials, clinical record numbers and other identifying information were anonymised. The study protocol was approved by hospital management and by the Research Ethics Committee of Aragón (C.P.–C.I. PI20/278).

## 3. Results

### 3.1. Descriptive Analysis

A total of 112 kidney transplant recipients were included in the study. The cohort was predominantly male (70.5%), with a median age of 58 years (IQR: 19.3; range: 18–80). Most participants (70.5%) were under 65 years of age. The leading causes of chronic kidney disease (CKD) were glomerulopathies (27.7%), followed by unknown aetiologies (21.4%), polycystic kidney disease (12.5%) and diabetic nephropathy (12.5%) (Table 1).

Regarding pre-transplant renal replacement therapy (RRT), 65.2% of participants had received haemodialysis, while 8.0% were transplanted without prior dialysis. Almost half had an arteriovenous fistula (44.6%), and the majority underwent their first transplantation (88.4%) with a deceased donor graft (89.3%). Concerning comorbidities, hypertension was present in 90.2% of the sample, dyslipidaemia in 52.7% and diabetes mellitus in 19.6%. Cardiovascular disease affected 26.0% of participants, and 18.0% reported toxic habits. When categorised by BMI, 47.3% of participants had normal weight, 34.0% were overweight and 14.3% were classified as obese. Men presented significantly higher BMI values than women (*p* = 0.007) (Table 2).

Median waiting time was 326 days (IQR: 506), being significantly longer in those <65 years (387 vs. 194 days; *p* = 0.010). Biochemical markers at baseline revealed elevated ferritin, urea and creatinine, with improvements in renal function and haemoglobin levels at 12 months. Statistically significant sex differences in creatinine levels were observed at both baseline (*p* = 0.016) and follow-up (*p* = 0.001) (Table 3).

### 3.2. Body Composition Changes over Time

Significant changes were observed over the follow-up period in several anthropometric and body composition parameters. Repeated-measures analyses revealed statistically significant differences in body weight, muscle mass, fat mass, and visceral fat index (all *p* < 0.05), while no significant differences were found in total body water percentage. Body weight showed a progressive increase over time (F(1,94) = 18.15, *p* < 0.001, η^2^ = 0.141, power = 1). Bonferroni-adjusted post hoc comparisons indicated significant increases from baseline to 6 months (mean difference = −2.36 kg, *p* < 0.001, 95% CI −3.75 to −0.95) and to 12 months (−3.24 kg, *p* < 0.001, 95% CI −4.96 to −1.51). A smaller but significant change was also noted between 3 and 12 months (−2.01 kg, *p* < 0.001) (Table 4).

**Figure 1:**
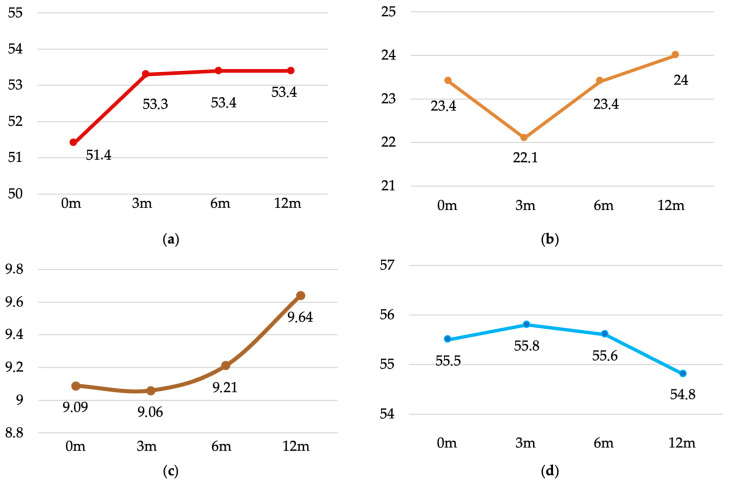
Evolution of muscle mass, fat mass, visceral fat and body water: (**a**) Muscle mass evolution (in kg). (**b**) Fat mass evolution (in percentage). (**c**) Visceral mass evolution (as index). (**d**) Body water evolution (in percentage).

Muscle mass followed an initial increase at 3 months, stabilising thereafter. Differences were significant across all time points (F(2,62) = 13.56, *p* < 0.001, η^2^ = 0.109), with post hoc tests confirming changes from baseline to 3, 6 and 12 months (all *p* < 0.001) (Figure 1a). Fat mass decreased during the first three months and then slightly increased between months 6 and 12. Overall differences were significant (F(2,64) = 4.97, *p* = 0.004, η^2^ = 0.043), with post hoc comparisons revealing changes from 3 to 6 months (−1.32%, *p* = 0.013) and from 3 to 12 months (−1.97%, *p* = 0.001) (Figure 1b). Visceral fat decreased slightly at 3 months and then increased over the following months (F(2,71) = 3.38, *p* = 0.023, η^2^ = 0.013). Significant changes were observed between 3 and 12 months (−0.58, *p* = 0.024) (Figure 1c). Total body water showed a modest increase at 3 months, followed by a gradual decline to below baseline values by 12 months, although these differences did not reach statistical significance (F(2,69) = 1.61, *p* = 0.190) (Figure 1d).

#### 3.2.1. Body Weight Changes over Time

Table 5 summarises the evolution of body weight according to the main clinical and demographic variables. Across all follow-up periods, men consistently showed higher body weight than women, with statistically significant differences at each time point (*p* < 0.001). Although men exhibited greater absolute weight gain, this difference is partly explained by their higher baseline weight.

Patients younger than 65 years also showed greater increases than older ones, with significant intra-group differences in the younger group (*p* < 0.001). According to previous kidney replacement therapy, non-dialysis patients gained more weight, while haemodialysis recipients showed smaller but still significant increases (*p* < 0.001). Regarding baseline BMI, individuals with underweight, normal weight or overweight showed significant increases, whereas those with obesity exhibited minimal changes. The most pronounced effect was observed in patients with a previous transplant, who gained on average 8.6 ± 4.6 kg compared with 2.5 ± 6.8 kg in first transplants (*p* = 0.008). Finally, recipients of a living donor kidney exhibited a higher early gain than those with a cadaveric donor (5.3 ± 4.0 vs. 3.0 ± 7.0 kg; *p* < 0.001).

#### 3.2.2. Evolution of Muscle Mass (MM) According to Independent Variables

Men showed a greater increase in muscle mass compared with women, with statistically significant differences at all measurement points (*p* < 0.001). Age group did not reveal differences between patients ≤ 65 and ≥65 years, although both groups experienced a significant intra-group increase in muscle mass over time (*p* < 0.001). Regarding KRT, haemodialysis patients presented the greatest increase, with significant intra-group differences (*p* < 0.001), whereas non-dialysis patients also showed an increase (*p* = 0.018) and peritoneal dialysis patients remained stable. Patients with previous transplantation gained more muscle mass than those undergoing their first transplant, but between-group differences were not significant; however, intra-group increases were observed in both groups (*p* < 0.001 and *p* = 0.043, respectively). Finally, recipients of cadaveric donor kidneys showed a significant increase over follow-up (*p* < 0.001), while those receiving a living donor kidney did not show statistically significant changes (Table 6).

#### 3.2.3. Evolution of Fat Mass (FM) According to Independent Variables

Women showed a significantly greater increase in FM compared to men, with differences already evident at 3 months (*p* = 0.018) and persisting at 6 and 12 months (*p* < 0.001). Intra-group analysis revealed significant changes only in men (*p* = 0.014). When stratified by age, patients under 65 years experienced an increase in FM (1.2%), while those aged 65 and older showed a slight decrease (−0.7%), with intra-group differences observed in the younger group (*p* = 0.018). According to KRT modality, FM changes were greater in non-dialysis and PD patients (2%) than in HD patients (0%), although no between-group or intra-group differences were identified. Previous transplantation was associated with a higher increase in FM (2.8%) compared with first transplantation (0.4%), without between-group differences; however, intra-group differences were significant only in patients without previous transplantation (*p* = 0.010). Finally, LDKT recipients showed a higher increase in FM (3.8%) compared to cadaveric donors (0.3%), with significant intra-group differences in both groups (Table 7).

#### 3.2.4. Evolution of Visceral Fat (VF) According to Independent Variables

Women experienced a greater increase in VF than men, with statistically significant differences at 3 and 12 months (*p* = 0.024 and *p* = 0.031, respectively), although intra-group changes were only significant in men (*p* = 0.018). According to age, patients under 65 years showed a higher increase in VF, with intra-group significance (*p* = 0.002), while those over 65 years exhibited a more stable trend without significant differences. Regarding KRT, HD patients presented the highest VF increase, with significant intra-group changes (*p* < 0.001), while no significant changes were observed in non-dialysis or PD groups. Patients with a previous transplant also showed greater VF gain, but significant intra-group changes were observed only in those undergoing first transplantation (*p* < 0.001). With respect to donor type, recipients of a cadaveric kidney presented significant intra-group increases in VF (*p* < 0.001), whereas no significant changes were observed in LDKT recipients (Table 8).

#### 3.2.5. Evolution of Total Body Water Percentage (%TBW) According to Independent Variables

In men, %TBW remained relatively stable throughout follow-up, with only minor intra-group variations, while women showed a progressive decrease, reaching statistically significant intra-group differences (*p* < 0.001). According to age, patients under 65 years showed a decrease in %TBW, significant in intra-group analysis (*p* < 0.001), whereas older patients maintained more stable values. By KRT modality, HD patients exhibited significant intra-group reductions in %TBW (*p* < 0.001), while no changes were detected in PD or non-dialysis patients. In patients with previous transplantation, %TBW remained relatively stable, while those with a first transplantation showed a significant intra-group reduction (*p* < 0.001). Regarding donor type, cadaveric transplant recipients presented significant intra-group decreases in %TBW (*p* < 0.001), whereas no relevant differences were observed in LDKT recipients (Table 9).

#### 3.2.6. Weight Gain According to Comorbidities

The analysis of weight gains according to comorbidities revealed that hypertensive patients exhibited a higher increase in body weight compared with non-hypertensive individuals, although the difference did not reach statistical significance. Similarly, those with a history of ischaemic heart disease showed greater weight gain than those without, but no significant between-group differences were observed. In contrast, patients without a history of dyslipidaemia, diabetes mellitus, respiratory disease or cerebrovascular accident experienced greater weight gain than their counterparts, although none of these comparisons reached statistical significance. Of the patients with pre-transplant diabetes and those who developed de novo diabetes after transplantation (*n* = 44), 5 patients were taking sodium–glucose cotransporter 2 (SGLT2 inhibitors), and 4 patients were taking dipeptidyl peptidase-4 inhibitors at the end of the study. Notably, patients who developed New-Onset Diabetes After Transplantation (NODAT) gained less weight compared with those who maintained normoglycaemia, with statistically significant differences (*p* = 0.013), only 2 patients of this group were taking SGLT2 inhibitors.

Furthermore, smokers experienced a significantly higher weight gain than non-smokers (*p* = 0.011) (Table 10).

At the end of the study, the overall survival rate of kidney transplant patients was 96.4%, with four events occurring during the follow-up period due to various causes unrelated to the kidney transplant, establishing an average survival rate of 1202.14 ± 13.70 days, [95% CI: 1175.28–1230]. The overall survival rate of grafts was 93.8%, with an average survival time of 1177.79 ± 19.07 days [95% CI: 1140.40–1215.18].

## 4. Discussion

### 4.1. Sociodemographic Characteristics

In this cohort, nearly three quarters of the participants were male, a distribution consistent with most epidemiological studies on CKD [30,31]. Biological sex plays a well-recognised role in CKD onset and progression: men present a higher incidence of end-stage kidney disease and CKD-related mortality, whereas women typically show a slower decline in renal function [32,33]. These differences appear to be primarily biological rather than sociocultural, influenced by genetic, hormonal, and environmental mechanisms [34,35]. Despite this biological advantage, women face systemic inequities in access to renal replacement therapies: they are more likely to donate a kidney but less likely to be included on transplant waiting lists or to receive a graft, particularly from living donors [36,37]. Our findings reflect these trends, highlighting the intersection of biological sex and gender-related disparities in kidney transplantation.

The proportion of recipients aged ≥65 years was higher than typically reported in similar series, reflecting broader eligibility criteria and improved management of comorbidities in recent years [38]. Although advanced age increases the complexity of transplantation, it is no longer considered an exclusion factor, and favourable outcomes have been reported in carefully selected older candidates [39,40]. Glomerulopathies emerged as the leading cause of CKD in this cohort, in line with both national and international data [41,42]. Most transplants were from deceased donors, with a relatively low proportion of living donor procedures compared to other countries with comparable healthcare systems [43,44], suggesting potential areas for improvement in promoting living donation.

### 4.2. Cardiovascular Risk Factors

Hypertension was the most prevalent cardiovascular risk factor, followed by dyslipidaemia and established cardiovascular disease in nearly one quarter of the patients. These findings confirm the substantial burden of cardiovascular comorbidity in kidney transplant recipients, although the prevalence observed was slightly lower than previously reported in Spanish cohorts [30]. This may reflect regional variations in patient selection, perioperative management, or cardiovascular risk assessment strategies. Given the central role of cardiovascular disease in post-transplant morbidity and mortality, our data underscore the importance of structured cardiovascular monitoring both before and after transplantation.

### 4.3. Weight Evolution

At 12 months post-transplant, patients in this cohort gained on average 3.6 kg (5.1% of baseline weight). This increase is more modest than the 8–15% reported in previous national and international studies [1,3]. The wide variability in reported trajectories, ranging from significant weight gain to clinically relevant weight loss [45], highlights the heterogeneous metabolic responses to transplantation. Importantly, both marked weight gain and weight loss have been associated with higher mortality compared to weight-stable recipients [46], emphasising the need for close post-transplant monitoring.

In our sample, a subset of patients experienced weight loss during follow-up. This finding, though less frequently reported, may reflect increased metabolic demands, immunological adaptation, or nutritional imbalances. While moderate weight reduction could represent a favourable metabolic shift, unintended or excessive loss may compromise recovery and indicate subclinical complications [47]. These results reinforce the clinical importance of monitoring not only weight gain, but also unexpected reductions in body weight.

Weight trajectories also differed by sex. Male recipients exhibited greater weight gain, a trend aligned with studies documenting higher overweight and obesity rates among male kidney transplant recipients [48]. However, other reports have shown higher increases in female cohorts [49], suggesting that post-transplant weight change is shaped by both biological and behavioural determinants. Gender-specific differences in diet and physical activity behaviours may partly explain inconsistencies in post-transplant weight trajectories. In kidney transplant cohorts, adherence to healthy diets—such as the Mediterranean diet—is generally suboptimal, and nearly one-third of recipients report low levels of physical activity. Moreover, women often report better compliance with dietary recommendations and higher levels of physical activity than men, but the metabolic benefits may be attenuated by hormonal or genetic factors [50,51].

Age also emerged as a determinant of weight change, with younger recipients showing greater gains compared to older patients, in agreement with previous studies [52,53]. This pattern may be explained by a more cautious dietary approach among older patients—often motivated by comorbidities—whereas younger recipients are more likely to relax dietary restrictions and increase carbohydrate intake after transplantation [54]. Nonetheless, age-related metabolic and hormonal changes can also predispose to weight gain, suggesting that the relationship between age and post-transplant weight is complex and potentially bidirectional [55].

Overall, our findings suggest that weight trajectories after kidney transplantation are influenced by a combination of biological, behavioural, and age-related factors. These observations highlight the importance of personalised nutritional counselling and lifestyle interventions, particularly in younger and male recipients, who appear more prone to excessive post-transplant weight gain.

### 4.4. Body Composition Changes

Beyond changes in body weight, this study provides a detailed assessment of body composition, offering insights into the qualitative nature of post-transplant weight trajectories. The findings of this study evidenced an increase lean mass during the first three months post-transplant, which remained stable throughout the follow-up period. This contrasts with other study that reported an increase in total body weight without a corresponding increase in muscle mass [56]. Not having to go to hospital (65.2% of patients underwent haemodialysis) can result in patients enjoying more free time outside the home and the elimination of dietary restrictions. Together with cultural, environmental and social differences, this could explain the significant improvement in muscle mass in our patients. This phenomenon is particularly relevant as loss of muscle mass—sarcopenia—has been associated with impaired functional capacity, increased frailty, and worse survival outcomes in this population [57].

Differences in lean mass according to sex indicate a greater gain in males, a finding that is consistent with the results reported in the existing literature. These sex-specific trajectories may reflect differences in hormonal status, and behavioural adaptation., fat distribution. While men are more likely to accumulate visceral fat and lose muscle mass, women tend to maintain peripheral fat deposits [10,58]. Nonetheless, both patterns have important clinical implications: visceral adiposity is linked to cardiometabolic risk, whereas sarcopenia predisposes to frailty and physical dependence.

Age also emerged as a modifier of body composition. Older recipients displayed a relative preservation of fat-free mass compared with younger patients, who showed greater increases in fat accumulation. This may be explained by stricter adherence to dietary recommendations and more stable lifestyle behaviours among older adults, while younger recipients are more prone to overnutrition, and sedentary habits once dietary restrictions are relaxed post-transplant [54]. However, the combination of age-related anabolic resistance and immunosuppressive therapy can exacerbate sarcopenic changes, suggesting that older patients may still be at risk for progressive muscle decline despite stable weight [23].

The hydration status is consistent with previous findings reporting redistribution of fluid compartments following transplantation [59]. Changes in total body water and extracellular water may be influenced by immunosuppressive regimens, graft function, and concomitant comorbidities [60]. These shifts have prognostic significance, as fluid overload is a recognised predictor of cardiovascular morbidity and mortality in this population [61,62].

Taken together, these results reinforce the notion that body composition, rather than weight alone, provides a more accurate picture of the nutritional and functional status of kidney transplant recipients. Routine incorporation of body composition assessment in clinical practice could enable early identification of patients at risk of sarcopenia, adiposity-related complications, or fluid overload, allowing for tailored interventions.

### 4.5. Clinical Implications

The findings of this study underscore the need for a comprehensive and individualised approach to the nutritional and metabolic follow-up of kidney transplant recipients. While body weight monitoring remains routine, assessment of body composition provides additional insights into the qualitative nature of weight change and its impact on patient outcomes. Early detection of sarcopenia, excessive fat gain, or fluid overload may facilitate targeted interventions, such as personalised dietary counselling, structured physical activity programmes, and optimisation of immunosuppressive regimens. Incorporating body composition monitoring into post-transplant care could improve the long-term health, functionality, and survival of this vulnerable population.

From a preventive standpoint, identifying subgroups at higher risk—such as younger recipients, men, and patients with pre-existing cardiovascular risk factors—may allow for the development of tailored educational and lifestyle interventions. Moreover, the increasing proportion of older transplant recipients highlights the need to balance functional preservation with adequate nutritional support, avoiding both sarcopenia and excessive weight gain.

### 4.6. Strengths and Limitations

This study presents several limitations that should be considered when interpreting the findings. First, it was conducted in a single tertiary hospital, which may limit the generalisability of results to other regions with different clinical practices or sociodemographic characteristics. Second, lifestyle-related variables such as physical activity, employment or retirement status, and adherence to dietary recommendations were not assessed. Similarly, functional capacity metrics, immunosuppressive medication, risk of infections, hospitalizations, patient and graft survival and its relationship to changes in body composition were not assessed either, despite their recognised influence on post-transplant weight and body composition trajectories. Third, although body composition was measured using bioelectrical impedance analysis, a widely available and non-invasive method, this technique is less precise than gold-standard approaches such as dual-energy X-ray absorptiometry or magnetic resonance imaging and may be influenced by hydration status. Finally, the follow-up period was limited to 12 months, precluding the analysis of long-term changes and their association with graft survival and overall patient prognosis.

Despite these limitations, the study has several important strengths. Its prospective design allows for a temporal assessment of changes in body composition during the first year after kidney transplantation. Furthermore, the study included all transplant recipients in the reference autonomous community, ensuring that the cohort was highly representative of the target population. The relatively large sample size and the inclusion of a broad age spectrum, with nearly one third of participants over 65 years, enhance the applicability of the findings to contemporary clinical practice. Lastly, the use of body composition analysis provided a more accurate evaluation of fat, lean and fluid compartments compared to traditional measures such as body mass index, offering a more nuanced understanding of post-transplant nutritional and metabolic status.

## 5. Conclusions

The findings of this prospective cohort study indicate that post-transplant changes in body composition are strongly influenced by demographic and clinical variables. Differences were observed in all measures, including body weight, lean mass, fat mass and visceral adiposity. Both sex and baseline BMI emerged as key determinants, highlighting the importance of individualised monitoring strategies.

Older recipients (>65 years) exhibited greater overall increases in body weight and adiposity, whereas younger patients accumulated proportionally more visceral fat, a distribution pattern associated with higher metabolic risk. The development of new-onset diabetes after transplantation was paradoxically associated with less weight gain, suggesting complex metabolic adaptations that warrant further investigation. In addition, active smoking was linked to a greater increase in body weight, underscoring the need for targeted preventive counselling in this subgroup.

Overall, these findings emphasise the relevance of systematic and early assessment of body composition in kidney transplant recipients, beyond traditional reliance on BMI. Regular monitoring may facilitate the timely identification of patients at higher risk of adverse metabolic trajectories, allowing for the implementation of tailored dietary, lifestyle and pharmacological interventions. Future research should extend the follow-up to longer periods, evaluate the role of physical activity and nutritional adherence, and explore the impact of these changes on graft function, cardiovascular risk and long-term survival.

## Figures and Tables

**Table 1:** Distribution of CKD.

Cause	*n* (%)
Glomerulopathies	31 (27.70)
Unaffiliated	24 (21.40)
Diabetes mellitus	14 (12.50)
Polycystic kidney disease	14 (12.50)
Tubulo-interstitial	12 (10.70)
Hypertension	8 (8.04)
Metabolic-congenital	5 (4.46)
Renovascular	3 (2.70)
Total	112 (100)

**Table 2:** Baseline sociodemographic and clinical characteristics.

		*n* (%)	Men	Women	*p*
		112 (100%)	79 (70.5%)	33 (29.5%)	-
Age (categorised)	18–64	79 (70.5)	57 (72.2)	22 (66.7)	0562 ^x^
	≥65	33 (29.5)	22 (27.8)	11 (33.3)	
Kidney replacement therapy	Non-dialysis	9 (8.1)	6 (7.6)	3 (9.1)	0.784 ^+^
	Peritoneal dialysis	30 (26.8)	23 (29.1)	7 (21.2)	
	Haemodialysis	73 (65.2)	50 (63.3)	23 (69.7)	
Transplant Previous	No	99 (88.4)	71 (89.9)	28 (84.8)	0.449 ^x^
	Yes	13 (11.6)	8 (10.1)	5 (15.2)	
Vascular Access	No vascular Access	39 (34.8)	29 (36.7)	10 (30.3)	0.255 ^+^
	AVF	50 (44.6)	37 (46.8)	13 (39.4)	
	Catheter	23 (20.5)	13 (16.5)	10 (30.3)	
BMI	Under weight	5 (4.5)	1 (1.3)	4 (12.1)	0.007 ^+^
	Normal weight	53 (47.3)	34 (43.0)	19 (57.5)	
	Overweight	38 (34.0)	33 (41.8)	5 (15.2)	
	Obesity	16 (14.3)	11 (13.9)	5 (15.2)	
Patients with hypertension		101 (90.2)	71 (70.3)	30 (29.7)	0.867 ^x^
Patients with dyslipidaemia		59 (52.7)	43 (72.9)	16 (27.1)	0.566 ^x^
Patients with previous diabetes		22 (19.6)	16 (72.7)	6 (27.3)	0.801 ^x^
Patients with Ischemic Heart disease		29 (26.0)	21 (26.6)	8 (24.2)	0.797 ^x^
Patients with respiratory disease		15 (13.4)	14 (17.7)	1 (3.0)	0.037 ^x^
Patients with cerebrovascular accident		5 (4.5)	3 (29.0)	2 (40.0)	0.597 ^x^
Patients with toxic habit		20 (18.0)	16 (80.0)	4 (20.0)	0.306 ^x^

AVF: Arteriovenous fistula; ^x^ Chi-square; ^+^ Fisher’s Test; T Student’s *t* test; U: Mann–Whitney’s U test.

**Table 3:** Blood parameters.

Variable	Pre-Transplant				12 Months			
	Total	Men	Women	*p*	Total	Men	Women	*p*
Ferritin (15–200 ng/mL)	384.85	355	387	0.632 ^U^	335	287.70	381	0.390 ^U^
	(445.20)	(467)	(362)		(550.40)	(502)	(636)	
Urea (17–43 mg/dL)	107.00	112.00	99.00	0.165 ^U^	58	60	54	0.278 ^U^
	(80.00)	(79.50)	(69)		(28.50)	(28.50)	(31.0)	
Creatinine (0.51–0.95 mg/dL)	6.08	6.21	5.40	0.016 ^U^	1.48	1.54	1.25	0.001 ^U^
	(3.03)	(3.78)	(2.83)		(0.66)	(0.57)	(0.59)	
Total. protein (6.6–8.3 g/dL)	6.60	6.70	6.60	0.196 ^T^	6.71	6.70	6.80	0.856 ^T^
	(0.80)	(0.60)	(0.61)		(0.80)	(0.750	(0.80)	
Albumin (3.5–5.2 g/dL)	4.00	4.03	3.90	0.128 ^T^	4.20	4.30	4.20	0.279 ^T^
	(0.60)	(0.60)	(0.60)		(0.80)	(0.50)	(0.40)	
Haemoglobin (g/dL)	11.90	12.00	11.7	0.380 ^T^	13.40	13.50	12.90	0.538 ^T^
	(1.92)	(1.90)	(2.10)		(2.075)	(1.75)	(2.50)	
Haematocrit (%)	35.65	35.9	35.50	0.699 ^T^	39.90	40.30	38.70	0.791 ^T^
	(5.12)	(4.90)	(5.70)		(6.20)	(5.60)	(0.94)	
Glycated haemoglobin (HbA1c)	5.30	5.20	5.30	0.924 ^U^	5.80	5.81	5.80	0.897 ^U^
	(0.71)	(0.70)	(0.80)		(1.15)	(1.19)	(0.95)	

Data are shown as median (interquartile range); ^T^: Student’s *t* test; ^U^: Mann–Whitney’s U test.

**Table 4:** Distribution of body composition over time periods.

Variable	0 Months	3 Months	6 Months	12 Months	*p*-ANOVA
Total weight (kg)	71.3 ± 15.5	72.5 ± 15.1	73.7 ± 15.0	74.5 ± 15.2	<0.001
	(68.4–74.2)	(69.7–75.4)	(70.8–76.5)	(71.7–77.4)	
Muscle Mass (kg)	51.4 ± 10.7	53.3 ± 11.1	53.4 ± 11.1	53.4 ± 11.0	<0.001
	(49.4–53.4)	(51.2–55.3)	(51.3–55.5)	(51.3–55.4)	
Fat mass (%)	23.4 ± 9.0	22.1 ± 8.8	23.4 ± 8.8	24.1 ± 9.0	0.004
	(21.7–25.1)	(20.5–23.8)	(21.8–25.1)	(22.4–25.8)	
Visceral Fat (index)	9.09 ± 4.77	9.06 ± 4.37	9.21 ± 4.51	9.64 ± 4.47	0.023
	(8.20–9.98)	(8.24–9.88)	(8.36–10.05)	(8.81–10.48)	
Body water (%)	55.5 ± 7.4	55.8 ± 7.3	55.6 ± 6.9	54.8 ± 6.6	0.190
	(54.1–56.8)	(54.4–57.2)	(54.3–56.9)	(53.6–56.0)	

**Table 5:** Weighting in time periods according to qualitative variables.

Time Periods		0 Months		3 Months		6 Months		12 Months		*p*-ANOVA
Variable		Weight	*p*	Weight	*p*	Weight	*p*	Weight	*p*	
Sex	Men	75.3 ± 13.8 (72.3–78.4)	<0.001	76.4 ± 13.8 (73.3–79.5)	<0.001	77.7 ± 13.9 (74.5–80.8)	<0.001	78.7 ± 13.8 (75.6–81.7)	<0.001	<0.001
	Women	61.6 ± 15.4 (56.1–67.1)		63.2 ± 14.0 (58.2–68.1)		64.1 ± 13.5 (59.3–68.9)		64.7 ± 14.1 (59.7–69.7)		0.002
Age	≤65 y.	71.7 ± 16.0 (68.1–75.3)	0.726	72.8 ± 14.9 (69.4–76.1)	0.954	74.0 ± 14.9 (70.7–77.4)	0.688	75.3 ± 15.0 (71.9–78.6)	0.403	<0.001
	≥65 y.	70.3 ± 14.5 (65.1–75.4)		71.9 ± 15.7 (66.4–77.5)		72.7 ± 15.6 (67.2–78.3)		72.8 ± 15.8 (67.2–78.4)		0.066
KRT	Non- dialysis	68.8 ± 15.9 (56.6–81.0)	0.515	71.0 ± 16.4 (58.4–83.6)	0.631	71.5 ± 16.2 (59.0–84.0)	0.639	73.1 ± 16.3 (60.6–85.6)	0.662	0.004
	PD	75.0 ± 17.9 (68.3–81.7)		75.3 ± 16.4 (69.2–81.5)		76.4 ± 16.6 (70.2–82.6)		77.1 ± 16.6 (70.9–83.3)		0.082
	HD	70.1 ± 14.4 (66.7–73.5)		71.6 ± 14.5 (68.2–74.9)		72.8 ± 14.3 (69.5–76.1)		73.7 ± 14.6 (70.3–77.0)		<0.001
Vascular Access	None	73.3 ± 17.6 (67.6–78.9)	0.074	73.9 ± 16.4 (68.6–79.2)	0.172	74.8 ± 16.6 (69.5–80.2)	0.250	75.7 ± 16.6 (70.3–81.1)	0.380	0.003
	AVF	72.6 ± 12.8 (68.9–76.2)		73.8 ± 13.4 (70.0–77.6)		74.7 ± 13.5 (70.9–78.6)		75.5 ± 13.9 (71.6–79.5)		0.006
	Catheter	65.2 ± 16.5 (58.1–72.3)		67.4 ± 15.9 (60.5–74.3)		69.3 ± 15.3 (62.7–75.9)		70.4 ± 15.4 (63.7–77.0)		0.003
BMI	Under weight	46.6 ± 4.7 (40.7–52.4)	<0.001	49.5 ± 5.5 (42.6–56.3)	<0.001	50.6 ± 6.1 (43.0–58.1)	<0.001	50.4 ± 5.0 (44.1–56.6)	<0.001	0.009
	Normal weight	63.9 ± 11.0 (60.8–66.9)		65.4 ± 10.9 (62.4–68.4)		66.6 ± 10.6 (63.7–69.5)		67.5 ± 11.0 (64.5–70.6)		<0.001
	Over weight	76.9 ± 11.0 (73.2–80.5)		78.2 ± 10.6 (74.7–81.7)		79.3 ± 10.8 (75.8–82.9)		80.5 ± 10.0 (77.2–83.8)		<0.001
	Obesity	90.5 ± 13.7 (83.2–97.8)		89.8 ± 15.3 (81.6–98.0)		90.7 ± 15.7 (82.3–99.0)		91.1 ± 17.0 (82.0–100.1)		0.759
Previous transplant	No	71.9 ± 15.1 (68.9–74.9)	0.361	72.9 ± 14.7 (70.0–75.9)	0.540	73.8 ± 14.5 (70.9–76.7)	0.835	74.5 ± 19.5 (62.7–86.3)	0.892	<0.001
	Yes	66.7 ± 18.4 (55.5–77.8)		69.5 ± 18.1 (58.6–80.5)		72.9 ± 19.1 (61.3–84.4)		75.3 ± 19.4 (62.9–87.6)		<0.001
Type donor	Living	70.0 ± 21.0 (56.7–83.4)	0.799	72.8 ± 20.4 (59.8–85.7)	0.899	73.9 ± 19.6 (61.5–86.3)	0.944	75.3 ± 19.4 (62.9–87.6)	0.925	<0.001
	Cadaver	71.5 ± 14.9 (68.5–74.4)		72.5 ± 14.5 (69.6–75.4)		73.6 ± 14.5 (70.7–76.5)		74.4 ± 14.7 (71.5–77.4)		0.000

Data are shown as mean ± SD and 95% CI; PD: peritoneal dialysis; HD: haemodialysis; AVF: arteriovenous fistula.

**Table 6:** Muscle mass (in kg) in the time periods according to qualitative variables.

Time Periods		0 Months		3 Months		6 Months		12 Months		*p*-ANOVA
Variable		Weight	*p*	Weight	*p*	Weight	*p*	Weight	*p*	
Sex	Men	55.5 ± 9.1 (53.4–57.5)	<0.001	57.7 ± 9.5 (55.6–59.8)	<0.001	57.8 ± 9.4 (55.7–59.9)	<0.001	57.9 ± 8.9 (55.9–59.8)	<0.001	<0.001
	Women	41.6 ± 7.4 (39.0–44.3)		42.7 ± 6.5 (40.4–45.0)		42.9 ± 7.4 (40.3–45.5)		42.7 ± 7.6 (40.0–45.4)		0.052
Age	≤65 y.	51.7 ± 10.9 (49.2–54.1)	0.695	53.2 ± 10.7 (50.8–55.6)	1	53.2 ± 10.6 (50.8–55.5)	0.962	53.6 ± 10.8 (51.1–56.0)	0.803	<0.001
	≥65 y.	50.7 ± 10.3 (47.1–54.4)		53.5 ± 12.1 (49.2–57.8)		54.1 ± 12.5 (49.6–58.5)		53.0 ± 11.5 (48.9–57.1)		<0.001
KRT	Non- dialysis	51.5 ± 12.8 (41.7–61.4)	0.730	54.6 ± 11.6 (45.6–63.5)	0.722	54.0 ± 12.3 (44.6–63.5)	0.865	53.2 ± 11.6 (44.3–62.1)	0.979	0.018
	PD	53.4 ± 11.4 (49.2–57.7)		54.4 ± 12.3 (49.8–59.0)		54.0 ± 12.0 (49.6–58.5)		53.9 ± 11.5 (49.6–58.2)		0.753
	HD	50.5 ± 10.2 (48.2–52.9)		52.6 ± 10.6 (50.1–55.1)		53.1 ± 10.8 (50.6–55.6)		53.2 ± 10.8 (50.7–55.7)		<0.001
Previous trasplant	No	51.9 ± 10.6 (49.7–54.0)	0.191	53.7 ± 11.2 (51.4–55.9)	0.388	53.6 ± 11.0 (51.4–55.8)	0.624	53.6 ± 10.9 (51.4–55.8)	0.465	<0.001
	Yes	47.9 ± 11.2 (41.1–54.6)		50.3 ± 10.1 (44.1–56.4)		52.1 ± 12.6 (44.5–59.7)		51.7 ± 11.9 (44.5–58.8)		0.043
Type donor	Living	50.9 ± 13.4 (42.3–59.4)	0.731	52.9 ± 12.7 (44.8–60.9)	0.836	53.1 ± 12.8 (44.9–61.3)	0.977	52.3 ± 11.7 (44.9–59.8)	0.550	0.108
	Cadaver	51.5 ± 10.4 (49.4–53.5)		53.3 ± 11.0 (51.1–55.5)		53.5 ± 11.0 (51.3–55.7)		53.5 ± 10.9 (51.3–55.7)		<0.001

Data are shown as mean ± SD and 95% CI; PD: peritoneal dialysis; HD: haemodialysis.

**Table 7:** Fat mass (in %) in the time periods according to qualitative variables.

Time Periods		0 Months		3 Months		6 Months		12 Months		*p*-ANOVA
Variable		Weight	*p*	Weight	*p*	Weight	*p*	Weight	*p*	
Sex	Men	21.9 ± 8.0 (20.1–23.7)	0.018	20.1 ± 7.6 (18.4–21.8)	0.001	21.4 ± 7.9 (19.6–23.2)	<0.001	21.9 ± 8.0 (20.1–23.7)	<0.001	0.014
	Women	27.1 ± 10.1 (23.5–30.7)		27.0 ± 9.5 (23.7–30.4)		28.3 ± 8.8 (25.2–31.5)		29.2 ± 9.1 (26.0–32.4)		0.069
Age	≤65 y.	23.3 ± 9.6 (21.1–25.4)	0.740	22.5 ± 9.2 (20.5–24.6)	0.391	23.9 ± 8.8 (21.9–25.9)	0.410	24.5 ± 9.6 (22.4–26.7)	0.505	0.018
	≥65 y.	23.7 ± 7.3 (21.1–26.3)		21.1 ± 7.7 (18.4–23.8)		22.3 ± 8.6 (19.3–25.4)		23.0 ± 7.2 (20.4–25.5)		0.053
KRT	Non- dialysis	20.9 ± 6.6 (15.9–26.0)	0.543	18.3 ± 6.5 (13.3–23.3)	0.216	20.1 ± 7.5 (14.4–25.9)	0.251	22.9 ± 7.0 (17.5–28.3)	0.447	0.050
	PD	24.0 ± 9.3 (20.5–27.5)		23.7 ± 9.1 (20.3–27.1)		25.2 ± 8.5 (22.0–28.4)		25.9 ± 9.8 (22.3–29.6)		0.159
	HD	23.5 ± 9.1 (21.3–25.6)		21.9 ± 8.8 (19.9–24.0)		23.1 ± 9.0 (21.0–25.2)		23.5 ± 8.8 (21.4–25.5)		0.069
Previous trasplant	No	23.5 ± 8.8 (21.7–25.2)	0.737	22.1 ± 8.8 (20.3–23.8)	1	23.4 ± 8.7 (21.7–25.1)	0.978	23.9 ± 8.8 (22.1–25.6)	0.650	0.010
	Yes	22.8 ± 10.8 (16.2–29.3)		22.6 ± 8.9 (17.2–28.0)		23.7 ± 9.3 (18.1–29.4)		25.6 ± 10.2 (19.4–31.8)		0.180
Type donor	Living	21.7 ± 9.1 (15.9–27.5)	0.528	22.2 ± 8.7 (16.7–27.8)	0.843	23.4 ± 9.6 (17.3–29.5)	0.825	25.5 ± 10.0 (19.1–31.9)	0.498	0.046
	Cadaver	23.6 ± 9.0 (21.8–25.4)		22.1 ± 8.8 (20.4–23.9)		23.4 ± 8.7 (21.7–25.2)		23.9 ± 8.9 (22.2–25.7)		0.010

Data are shown as mean ± SD and 95% CI; PD: peritoneal dialysis; HD: haemodialysis.

**Table 8:** Visceral mass (as index) in the time periods according to qualitative variables.

Time Periods		0 Months		3 Months		6 Months		12 Months		*p*-ANOVA
Variable		Weight	*p*	Weight	*p*	Weight	*p*	Weight	*p*	
Sex	Men	10.1 ± 4.8 (9.0–11.2)	0.001	9.9 ± 4.4 (9.0–10.9)	0.001	10.1 ± 4.7 (9.0–11.1)	0.001	10.6 ± 4.5 (9.6–11.6)	0.001	0.078
	Women	6.7 ± 3.7 (5.4–8.1)		6.9 ± 3.6 (5.7–8.2)		7.2 ± 3.3 (6.0–8.3)		7.3 ± 3.4 (6.1–8.5)		0.121
Age	≤65 y.	8.0 ± 4.5 (7.0–9.0)	0.001	8.0 ± 4.2 (7.1–9.0)	0.001	8.2 ± 4.4 (7.3–9.2)	0.001	8.7 ± 4.3 (7.7–9.7)	0.001	<0.001
	≥65 y.	11.8 ± 4.3 (10.3–13.3)		11.5 ± 3.6 (10.3–12.8)		11.5 ± 3.9 (10.1–12.9)		11.9 ± 4.0 (10.5–13.3)		0.751
KRT	Non- dialysis	7.2 ± 4.1 (4.1–10.3)	0.391	7.9 ± 4.5 (4.4–11.4)	0.641	8.1 ± 4.7 (4.5–11.7)	0.493	9.2 ± 5.0 (5.4–13.1)	0.779	0.012
	PD	9.7 ± 5.7 (7.5–11.8)		9.4 ± 5.1 (7.5–11.3)		10.1 ± 5.4 (8.1–12.1)		10.1 ± 5.3 (8.1–12.1)		0.323
	HD	9.1 ± 4.4 (8.1–10.1)		9.1 ± 4.1 (8.1–10.0)		9.0 ± 4.1 (8.0–9.9)		9.5 ± 4.1 (8.6–10.5)		0.160
Previous trasplant	No	9.2 ± 4.7 (8.3–10.1)	0.348	9.1 ± 4.3 (8.2–9.9)	0.740	9.2 ± 4.5 (8.3–10.1)	0.899	9.7 ± 4.5 (8.8–10.6)	0.841	0.003
	Yes	8.2 ± 5.3 (5.0–11.4)		8.8 ± 5.1 (5.8–11.9)		9.3 ± 5.2 (6.2–12.4)		9.5 ± 4.2 (6.9–1.0)		0.016
Type donor	Living	7.3 ± 5.8 (3.6–10.9)	0.134	8.1 ± 5.7 (4.5–11.7)	0.465	8.3 ± 5.6 (4.7–11.8)	0.534	8.4 ± 5.2 (5.1–11.7)	0.423	0.065
	Cadaver	9.3 ± 4.6 (8.4–10.2)		9.2 ± 4.2 (8.3–10.0)		9.3 ± 4.4 (8.5–10.2)		9.8 ± 4.4 (8.9–10.7)		0.001

Data are shown as mean ± SD and 95% CI; PD: peritoneal dialysis; HD: haemodialysis.

**Table 9:** Percentage of water in the time periods according to qualitative variables.

Time Periods		0 Months		3 Months		6 Months		12 Months		*p*-ANOVA
Variable		Weight	*p*	Weight	*p*	Weight	*p*	Weight	*p*	
Sex	Men	56.4 ± 7.3 (54.8–58.0)	0.067	57.6 ± 6.1 (56.3–59.0)	<0.001	57.1 ± 6.8 (55.6–58.6)	<0.001	56.3 ± 6.2 (54.9–57.7)	<0.001	0.013
	Women	53.2 ± 7.4 (50.5–55.8)		51.5 ± 8.3 (48.6–54.5)		52.0 ± 6.0 (49.8–54.1)		51.2 ± 6.1 (49.0–53.3)		0.096
Age	≤ 65 y.	55.5 ± 7.9 (53.7–57.2)	0.898	55.5 ± 7.3 (53.9–57.1)	0.431	55.0 ± 6.9 (53.4–56.5)	0.132	54.4 ± 7.0 (52.8–55.9)	0.285	0.007
	≥ 65 y.	55.4 ± 6.2 (53.2–57.7)		56.6 ± 7.5 (54.0–59.2)		57.0 ± 6.8 (54.6–59.5)		55.8 ± 5.5 (53.9–57.8)		0.043
KRT	Non- dialysis	56.7 ± 5.1 (52.8–60.7)	0.472	58.6 ± 6.0 (54.0–63.2)	0.240	57.0 ± 5.7 (52.6–61.4)	0.200	54.9 ± 5.4 (50.8–59.0)	0.445	0.087
	PD	54.8 ± 8.0 (51.8–57.8)		54.4 ± 8.6 (51.1–57.6)		54.3 ± 8.1 (51.3–57.3)		53.6 ± 7.4 (50.8–56.4)		0.596
	HD	55.6 ± 7.5 (53.8–57.3)		56.1 ± 6.9 (54–57.7)		55.9 ± 6.6 (54.4–57.5)		55.3 ± 6.4 (53.8–56.8)		0.062
Previous trasplant	No	55.3 ± 7.2 (53.9–56.7)	0.507	55.9 ± 7.5 (54.4–57.4)	0.867	55.6 ± 6.9 (54.3–57.0)	0.989	54.9 ± 6.5 (53.6–56.2)	0.696	0.013
	Yes	56.6 ± 9.4 (51.0–62.3)		55.5 ± 6.3 (51.6–59.3)		55.3 ± 7.4 (50.8–59.7)		54.1 ± 7.4 (49.6–58.6)		0.571
Type donor	Living	56.6 ± 7.4 (51.9–61.3)	0.410	56.1 ± 7.4 (51.4–60.8)	0.840	55.1 ± 7.2 (50.5–59.7)	0.724	53.8 ± 7.4 (49.1–58.5)	0.475	0.182
	Cadaver	55.3 ± 7.5 (53.8–56.8)		55.8 ± 7.4 (54.3–57.2)		55.7 ± 6.9 (54.3–57.0)		54.9 ± 6.5 (53.6–56.2)		0.013

Data are shown as mean ± SD and 95% CI; PD: peritoneal dialysis; HD: haemodialysis.

**Table 10:** Weight gain according to comorbidities.

Comorbidity		*n* (%)	Mean ± SD	CI 95%	*p*
Hypertension	No	11 (9.8)	2.11 ± 5.70	(−1.72–5.94)	0.564
	Yes	101 (90.2)	3.36 ± 6.90	(2.00–4.72)	
Dyslipidemia	No	53 (43.3)	4.07 ± 7.29	(2.06–6.08)	0.218
	Yes	59 (52.7)	2.48 ± 6.25	(0.85–4.11)	
Previous diabetes	No	90 (80.4)	3.35 ± 6.66	(1.95–4.74)	0.730
	Yes	22 (19.6)	2.79 ± 7.39	(−0.49–6.06)	
Ischemic heart disease	No	83 (74.1)	3.16 ± 6.83	(1.67–4.65)	0.843
	Yes	29 (25.9)	3.45 ± 6.76	(1.67–4.65)	
Respiratory disease	No	97 (86.6)	3.28 ± 6.73	(1.92–4.64)	0.860
	Yes	15 (13.4)	2.95 ± 7.34	(−1.12–7.01)	
Cerebrovascular accident	No	107 (95.5)	3.25 ± 6.92	(1.92–4.58)	0.916
	Yes	5 (4.5)	2.92 ± 2.84	(−0.60–6.44)	
NODAT ^1^	No	90 (80.4)	4.01 ± 6.54	(2.65–5.38)	0.013
	Yes	22 (19.6)	0.05 ± 6.97	(−3.04–3.14)	
Toxic habits	No	92 (82.1)	2.48 ± 6.47	(1.14–3.82)	0.011
	Yes	20 (17.9)	6.70 ± 7.27	(3.30–10.10)	

^1^ NODAT: New-Onset Diabetes After Transplantation.

## Data Availability

The data presented in this study are available upon request to the corresponding author due to confidentiality reasons.

## Abbreviations

The following abbreviations are used in this manuscript:

BIA	Bioelectrical Impedance Analysis
BMI	Body Mass Index
CI	Confidence Interval
CKD	Chronic Kidney Disease
CVD	Cardiovascular Disease
DLP	Dyslipidaemia
DM	Diabetes mellitus
FM	Fat mass
HD	Haemodialysis
HT	Hypertension
IQR	Interquartile Range
KRT	Kidney Replacement Therapy
LDKT	Living Donor Kidney Transplant
MM	Muscle Mass
NODAT	New Onset Diabetes After Transplantation
PD	Peritoneal Dialysis
PTDM	Post-Transplant Diabetes Mellitus
RRT	Renal Replacement Therapy
SD	Standard Deviation
SGLT2 Inhibitors	Sodium–glucose cotransporter 2
VF	Visceral Fat
WG	Weight Gain
WHO	World Health Organisation
%TBW	Total Body Water Percentage
